# Light Structures Phototroph, Bacterial and Fungal Communities at the Soil Surface

**DOI:** 10.1371/journal.pone.0069048

**Published:** 2013-07-19

**Authors:** Lawrence O. Davies, Hendrik Schäfer, Samantha Marshall, Irene Bramke, Robin G. Oliver, Gary D. Bending

**Affiliations:** 1 School of Life Sciences, Gibbet Hill Campus, University of Warwick, Coventry, United Kingdom; 2 Syngenta, Product Safety, Jealott’s Hill International Research Centre, Bracknell, Berkshire, United Kingdom; Uppsala University, Sweden

## Abstract

The upper few millimeters of soil harbour photosynthetic microbial communities that are structurally distinct from those of underlying bulk soil due to the presence of light. Previous studies in arid zones have demonstrated functional importance of these communities in reducing soil erosion, and enhancing carbon and nitrogen fixation. Despite being widely distributed, comparative understanding of the biodiversity of the soil surface and underlying soil is lacking, particularly in temperate zones. We investigated the establishment of soil surface communities on pasture soil in microcosms exposed to light or dark conditions, focusing on changes in phototroph, bacterial and fungal communities at the soil surface (0–3 mm) and bulk soil (3–12 mm) using ribosomal marker gene analyses. Microbial community structure changed with time and structurally similar phototrophic communities were found at the soil surface and in bulk soil in the light exposed microcosms suggesting that light can influence phototroph community structure even in the underlying bulk soil. 454 pyrosequencing showed a significant selection for diazotrophic cyanobacteria such as *Nostoc punctiforme* and *Anabaena* spp., in addition to the green alga *Scenedesmus obliquus*. The soil surface also harboured distinct heterotrophic bacterial and fungal communities in the presence of light, in particular, the selection for the phylum Firmicutes. However, these light driven changes in bacterial community structure did not extend to the underlying soil suggesting a discrete zone of influence, analogous to the rhizosphere.

## Introduction

The upper few millimeters of soil are an area with physicochemical conditions distinct from those of bulk soil as a result of the surface being exposed to light and other environmental factors such as wind and rain erosion [1]. Soil surface communities are different from those of bulk soil due to the development of photosynthetic communities such as cyanobacteria, algae, mosses, and lichens, which can form biological soil crusts (BSC) with time [2]–[7]. There has been a dramatic rise in publications reporting on the role of BSCs recently as it has become recognized that this area is a distinct ecosystem with increased nutrient levels [8] and erosion resistance [9] compared to soil without phototroph communities. BSC research to date has focused on arid and semi-arid lands such as the Colorado plateau and Sonoran desert in the USA [2]–[4], [10], Gurbantunggut desert in northwest China [7], [9], Negev desert in Israel [11] and Oman [6], where phototroph communities have been estimated to cover up to 70% of the soil surface [12]. BSCs have also been shown to be widespread in temperate soils and under agricultural crops [5], , however, little is known about their community structure and ecological significance.

In arid environments, soil surface communities have several important functions, including the release of exopolysaccharides from fungi and cyanobacteria which bind soil into aggregates, improving soil structure and reducing the impact of wind erosion [16]–[19]. Another key function of soil surface communities is fixation of N_2_ by diazotrophic cyanobacteria such as *Nostoc* spp. [4], [20]–[24], and C fixation by phototrophs [25]–[27], which may be the reason for higher soil C and N levels in soil with a BSC [8]. The development of phototrophs at the soil surface has also been shown to have a profound impact on plant growth and biomass [28] and result in increased levels of N, K, and Cu in plant tissues [29].

The development of BSC communities in arid environments is characterized by a succession from cyanobacteria dominated to lichen- and moss- dominated crusts [30]–[32]. Further, a succession within cyanobacteria dominated crusts has also been noted from *Microcoleus vaginatus* to *Nostoc* spp./*Tolypothrix* spp. [4]. However, our understanding of the community structure remains very limited, not least because the majority of studies investigating phototroph diversity in BSCs have used culture dependent methods which are prone to bias [5], [7], [9], [32], [33], or molecular methods that target 16S rRNA of bacteria, which ignore the diversity of eukaryotic phototrophs [2], [3], [6], [10], [11], [34]. Molecular microbial community analysis of bacterial diversity at the soil surface has shown a dominance by cyanobacteria [2], [3], [6], [11], for example, Abed *et al*. [6] found that 77–81% of clones from BSCs of Oman had close homology to cyanobacteria. Consequently, the diversity and community composition of heterotrophic bacteria at the soil surface is not well characterised. Likewise, although fungi have been shown to provide key ecosystem services of BSCs such as structural cohesion provided by hyphal entanglement [19], little is known regarding the fungal community structure at the soil surface [35], [36].

In contrast to arid and semi-arid soils, our understanding of the structure and function of soil surface communities in temperate and agricultural soils is limited [5], [28]. Phototrophs have been shown to develop under agricultural cropping systems such as wheat, maize and sugar beet between 50 and 80 days after tillage [15]. The presence of these communities reduced soil erosion rates and this reduction increased with the successional age of the crust [15]. However, soil tillage removed this functionally important community for at least 50 days [15]. Phototroph communities may also have other important agricultural functions, for example, several phototrophs have been shown to break down pesticides in pure culture [37] and therefore phototrophs may have a role in pesticide degradation at the soil surface. An understanding of the communities and functions of soil surface communities in temperate environments will inform agricultural management decisions such as the benefits of reduced tillage practices.

In this study, we investigated shifts in phototroph, bacterial and fungal community structure between the soil surface and bulk soil of a pasture soil from a temperate climate throughout the development of phototroph communities at the soil surface. We used universal phototroph primers designed to amplify ribosomal RNA genes of any plastid-containing organisms, 454 pyrosequencing of PCR amplicons, and measured soil pH and nutrient levels with the aim of answering the following questions: (i) How diverse are cyanobacteria and eukaryotic phototrophs at the soil surface? (ii) Does light influence bacterial and fungal community structure and diversity at the soil surface? (iii) Are there successional changes in phototroph, bacterial and fungal communities at the soil surface and underlying bulk soil? (iv) Does the establishment of soil surface communities affect chemical parameters and microbial community structure of underlying bulk soil?

## Materials and Methods

### Soil

Soil was sourced from Les Barges, Switzerland (CH-1896 Vouvry) in October, 2010. The site did not contain any protected wildlife and it is owned by Syngenta who authorized sampling. Approximately 40 kg was sampled from the top 15 cm of Gartenacker soil (silty loam), which was then sieved to 2 mm and homogenized by mixing to give an average representation of the community structure and chemical properties of the volume of sampled soil. Microbial communities in the upper 15 cm of soil are routinely disturbed and mixed by tillage. Soil was therefore sampled to this depth and homogenized before being setup in microscoms in order to simulate natural mixing of surface communities in agricultural systems. The land had been used for pasture for over 20 years without the application of pesticides. The physico-chemical properties of Gartenacker soil are shown in Table S1.

### Test System and Sampling Soil Surface Communities

To follow development of soil surface communities [Figure S1] a modified design was used from Jeffery *et al*. [1] with dimensions of 20 cm×15.5 cm×1.8 cm. Trays were filled with 600 g Gartenacker soil (35% water content) and soil was flattened to minimise soil surface heterogeneity. Trays were covered with either: (i) DS 226 light filter, or (ii) an opaque filter (Lee Filters, Andover, UK). In order to study the impact of light on microbial community development, soil was incubated in a controlled constant environment chamber on a 16 h:8 h light:dark cycle at 200 µmol s^−−1^ m^−1^ (Philips Master fluorescent lights (>360 nm) TLD 36 W/840) at a constant temperature of 20°C±2°C. This allowed the development of soil surface communities to be investigated under controlled conditions by removing confounding climatic variables. Trays were setup in triplicate using a randomised design; moisture content was checked weekly by weight and maintained by watering from above using a pipette.

Triplicate trays were destructively sampled at 0, 20, 40, and 80 days. This sampling strategy aimed to follow the development of early-successional phototroph communities based on previous work, which showed development of phototrophs under cropping systems between 50 and 80 days following tillage [15]. At each sampling point, a stainless steel sheet was run under the soil surface at a measured depth of 3 mm to separate the soil surface (upper 3 mm) from the underlying bulk soil (3–12 mm). Surface and bulk soil samples were frozen at −20°C in polyethylene zip bags for 48 h before freeze-drying for 72 h. Freeze-dried soil was homogenised using a mortar and pestle and stored at −20°C.

### Soil Chemical Properties

Extractable Mg and K were measured by adding 50 ml 1 M NH_4_NO_3_ solution to 10 g freeze-dried soil and shaking at 200 rpm for 30 mins. The solution was filtered prior to analysis using an ULTIMA 2 Inductively Coupled Plasma – Atomic Emission Spectroscopy (ICP-AES) (HORIBA Jobin Yvon, Middlesex, UK). Extractable nitrate (NO_3_) was measured by adding 50 ml saturated CaSO_4_ to 20 g freeze-dried soil and shaking at 200 rpm for 30 mins. The solution was filtered prior to colorimetric analysis using a FIAstar 5000 flow injection analyser (FOSS UK Ltd, Warrington, UK). Soil pH was measured by adding 25 ml water to 10 g freeze-dried soil and shaking at 200 rpm for 15 mins prior to pH measurement using an Accumet AR50 electrode (VWR, Leicestershire, UK). Extractable P was measured by adding 100 ml 0.5 M NaHCO_3_ solution (pH 8.5) to 5 g freeze-dried soil and shaking at 200 rpm for 30 mins. The solution was filtered prior to analysis by ICP-AES [38].

### Characterisation of Soil Surface Communities

#### Chlorophyll *a*

Chlorophyll *a* was extracted according to Ritchie [39]. Briefly, 20 ml 90% (v/v) acetone was added to 5 g freeze-dried soil and shaken at 300 rpm in the dark for 5 hours. Chlorophyll *a* was measured using a Shimadzu UV 1800 spectrophotometer at wavelengths 664 nm and 750 nm before acidifying with 3 M HCl for 90 seconds and re-measuring at 665 nm and 750 nm. Chlorophyll *a* values were calculated from the formulas given in Hansson [40].

#### Most probable number (MPN) of algae

At day 80, the number of algal cells at the soil surface under light and dark conditions was estimated using MPN. Fresh soil was homogenized and 1 g was transferred aseptically to 10 ml sterile Bold’s basal media (BBM) ([41]; method in Supporting Information S1). Serial dilutions were performed at 2, 4, 5, 6, 8, 10, 15, 20, and 25-fold dilutions and 5 replicates of 1 ml aliquots were transferred to a microtitre plate, covered with cling film and incubated for 21 days under a 16 h:8 h light:dark cycle at 200 µmol s^−1^ m^−1^. Algal growth was recorded by a colour change of BBM from clear to green. Algal abundance was estimated using a MPN calculator according to Blodgett [42].

### Microbial Community Structure at the Soil Surface

#### DNA extraction, PCR amplification of ribosomal RNA markers and Terminal Restriction Fragment Length Polymorphism (TRFLP) to assess phototroph, fungal and bacterial community structure

DNA was extracted using a FastDNA Spin Kit (Qbiogene, Loughborough, UK) according to the manufacturer’s handbook. The quantity and quality of DNA in extracts was analysed using a nanodrop ND-1000 spectrophotometer (Labtech International Ltd, Sussex, UK) and by agarose gel electrophoresis, respectively. DNA was extracted from surface and bulk soil samples after 0, 20, 40 and 80 days incubation under light and dark conditions.

The diversity of phototrophs was analysed by PCR targeting 23S rRNA genes of plastids using primers p23SrV_f1 and p23SrV_R1-HEX which produced a product approximately 410 bp in length [43]. Bacterial 16S rRNA genes were amplified using primers 63f and 1087r-VIC giving a 1 kb product [44], [45], and for analysis of fungi, PCR targeted the ITS region using primers ITS1f-PET and ITS4r [46], [47]. Details of all primer pairs are given in Table S2. PCR was performed using 47 µL MegaMix (Microzone Ltd, Haywards Heath, UK), 1 µL of DNA (10 ng/µL) and 1 µL of either 5 µM (bacteria/phototrophs) or 25 µM (fungi) forward and reverse primers. Samples were run on a GeneAmp 9700 thermocycler (Applied Biosystems, Warrington, UK) using the reaction described in Sherwood & Presting [43] for phototrophs. PCR amplification of 16S rRNA and the ITS region were run in the same reaction using the amplification method described by Marchesi *et al*. [45] with an extension time of 1 min and a final extension time of 10 mins (full methods are in Supporting Information S1).

PCR products were purified using a QIAquick PCR purification kit (Qiagen, Crawley, UK) according to the manufacturer’s instructions. Restriction digests were performed at 37°C for 4 hrs followed by 95°C for 15 mins. Digests of 23S rRNA gene fragments of phototrophs used 500 ng PCR product, 2 µL 10X buffer, 0.5 µL 5U *Dde*I (New England Biolabs, Hitchin, UK), made up to 20 µL with Ultra Pure DNase/RNase-free distilled water (Invitrogen, Paisley, UK). *Dde*I was used based on clone libraries using the Restriction Enzyme Mapping Application (REMA, http://bioperl.macaulay.ac.uk). Digests of 16S rRNA gene fragments of bacteria and ITS fragments of fungi used 500 ng and 400 ng of PCR product, respectively, 2 µL 10X buffer, 0.25 µL 5U of either *Msp*I or *Hha*I (New England Biolabs, Hitchin, UK), made up to 20 µL with sterilised distilled water. *Msp*I and *Hha*I were used as they have previously been shown to provide good differentiation between bacterial and fungal taxa [48]. Restriction digests using *Hha*I also contained 0.2 µL (10 mg/ml) bovine serum albumin (New England Biolabs, Hitchin, UK).

All samples were cleaned using Sephadex spin columns and LIZ1200 standard was added prior to electrophoresis using an ABI PRISM 3130×l genetic analyser (Applied Biosystems, Warrington, UK). GeneMarker (Softgenetics, USA) was used to quantify peak area of terminal restriction fragments (TRFs) and values were transformed to relative abundance to standardise data. A constant percentage threshold was selected according to Sait *et al*. [49] to minimise a correlation between total peak area and number of TRFs.

### 454 Amplicon Pyrosequencing to Determine Diversity of Phototrophs, Fungi and Bacteria at the Soil Surface

Phototroph, bacterial and fungal PCR amplicons from the soil surface incubated under light and dark conditions for 80 days were pyrosequenced by Research and Testing Laboratory (Lubbock, TX, USA) (RTL) using a Roche 454 FLX instrument and Titanium reagents. Bacterial Tag-encoded pyrosequencing was performed as described previously by Dowd *et al*. [50]. Fungal and phototroph pyrosequencing were performed according to the same protocol using the primer pairs described in the previous section. Pyrosequencing gave a total of 67658, 22672 and 77470 reads across six samples for bacteria, fungi and phototrophs, respectively.

### Processing of 454 Sequence Data

Sequences were processed using QIIME v. 1.4.0 [51] by selecting sequences with an average quality score >25, containing no ambiguous bases or homopolymers longer than six base pairs, without any primer mismatches, and a sequence length between 250–430 bp (bacteria), 250–390 bp (fungi) and 330–410 bp (phototrophs). Sequences were also denoised using Denoiser [52]. Following denoising, methods of data processing differed for bacteria, fungi and phototrophs. Bacterial OTUs were picked at a 97% similarity threshold using UCLUST [53] and representative sequences were picked using the most abundant method before PyNAST aligning [54] with the 16S rRNA Greengenes database aligned at 97% [55]. Chimeras were identified using ChimeraSlayer [56] and taxonomy was assigned using the RDP classifier and default settings [57]. Processing of fungi and phototrophs used UCHIME [58] for *de novo* chimera identification. Taxonomy was assigned using the RDP classifier for fungi [57] and BLAST [59] for phototrophs. Phylogenetic trees showing sequence abundance data were created using MEGAN 4 [60]. Full details of the number of sequences removed at each processing step are shown in Tables S3–S5. Sequence data have been submitted to the Genbank database under Bioproject Accession No. PRJNA179030.

### Statistical Analysis

Parametric tests on non-transformed data were performed where possible. If assumptions were not met, data was log transformed. One-way ANOVA was performed on chlorophyll *a*, pH and soil nutrient data, and t-tests were performed on MPN for algae and phototroph abundance data. All analyses were performed using Minitab version 15. TRF data was analysed using GeneMarker and statistically analysed using non-metric multidimensional scaling (NMDS) analysis, ANOSIM and SIMPER using PRIMER6 (Plymouth, UK). Pyrosequencing data was rarefied at 3317, 6322 and 964 reads for phototrophs, bacteria and fungi, respectively and QIIME v.1.4.0 was used for: ANOVAs to compare taxonomy abundance data and t-tests to compare α diversity. Chao1 was used as a mark-release-recapture assessment of diversity [61] and Observed Species as an assessment of the number of unique OTUs in a sample.

## Results

### Soil pH and Nutrients

Soil nutrient levels and pH are shown for all sampling points in Figures S2, S3, S4, S5, S6 and after 80 days incubation under light and dark conditions in Table 1. Light had a significant effect on pH, extractable NO_3_ and Mg (p≤0.001) at all sampling points (Figure S2, S3 & S5). At day 80, pH (p≤0.01) was higher and extractable NO_3_ (p≤0.01) and Mg (p≤0.01) were lower under light compared to dark incubated samples, however, there was no effect of depth (Table 1). Light did not influence extractable P, however, P was significantly higher at the soil surface compared to underlying bulk soil after 80 days incubation under light conditions (p≤0.01) (Table 1). Depth also influenced extractable K content with the soil surface having significantly higher extractable K than underlying bulk soil after 80 days incubation under light conditions (p≤0.01) (Table 1).

**Table 1:** The effect of light and depth on chlorophyll *a*, most probable number (MPN) of algae, pH, and extractable nitrate, phosphorus, potassium and magnesium after 80 days incubation under light and dark conditions (±1 standard error).

Treatment	Depth	MPN (cells g^−1^×10^3^)	pH	Nitrate(mg kg^−1^)	Phosphorous (mg kg^−1^)	Potassium (mg kg^−1^)	Magnesium (mg kg^−1^)
Light	Surface	69.15±6.5^a^	8.1±0.06^a^	6.3±0.3^a^	78.8±1.8^a^	104.2±5.1^a^	51.2±1.0^a^
	Bulk	Not measured	8.0±0.02^a^	4.3±2.2^a^	73.0±1.6^b^	76.2±3.6^b^	50.8±1.3^a^
Dark	Surface	1.08±0.15^b^	7.5±0.02^b^	74.1±1.3^b^	75.4±1.7^ab^	87.4±0.5^b^	60.8±1.8^b^
	Bulk	Not measured	7.5±0.01^b^	70.4±3.1^b^	75.6±0.6^ab^	85.4±2.7^b^	59.5±1.9^b^

Significant differences between treatments are indicated by different letters (p≤0.01).

### Most Probable Number for Algae and Chlorophyll a

**Figure 1:**
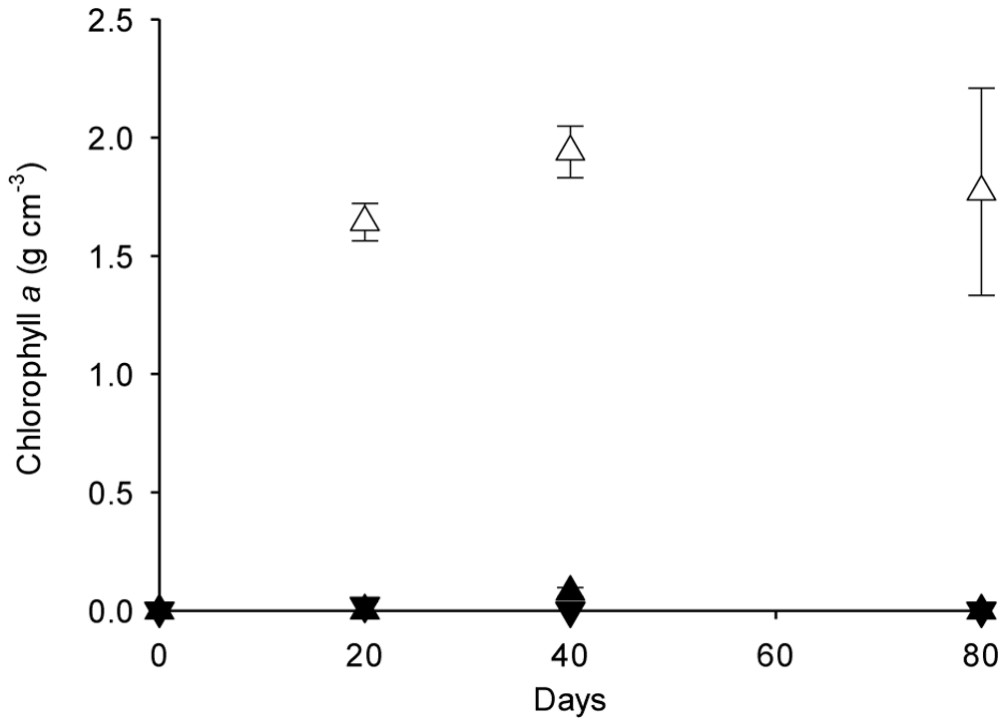
Chlorophyll *a* development in Gartenacker soil. Chlorophyll *a* in the surface (▴) and bulk (▾) of pasture soil after incubation under light (open symbols) or dark (closed symbols) conditions. Errors bars are ±1 standard error.

MPN assessment of algal abundance estimated a >60-fold greater algal population at the soil surface incubated under light compared to dark conditions for 80 days (p≤0.01) (Table 1). In addition, light (p≤0.001) and depth (p≤0.001) had a significant effect on chlorophyll *a* (Figure 1). Chlorophyll *a* was significantly higher at the soil surface under light at day 20, 40 and 80 (p≤0.001). Chlorophyll *a* was not detected in bulk soil under light or under dark conditions (Figure 1).

### TRFLP Analysis of Phototroph, Bacterial and Fungal Community Structure at the Soil Surface and Underlying Bulk Soil under Light and Dark Conditions

**Figure 2:**
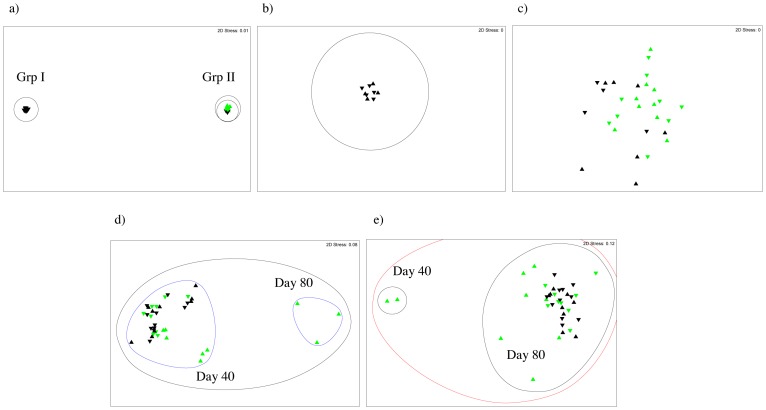
Development of phototroph, bacterial and fungal communities in Gartenacker soil. Phototroph (23S rRNA genes of plastids), bacterial (16S rRNA) and fungal (ITS) community structure at the surface (▴) and bulk (▾) of a pasture soil under light (green) and dark (black) conditions: (a) phototrophs all samples; (b) phototrophs close up of Grp I samples; (c) phototrophs close up of Grp II samples; (d) bacteria all samples (e) fungi all samples. Non-metric dimensional scaling shows clustering based on the similarity of microbial community structure between treatments: 15% (red cluster), 40% (black cluster) and 85% (blue cluster).

Phototroph community structure was significantly different at the soil surface (p≤0.01) and in bulk soil (p≤0.05) under light conditions compared to dark incubated soil (Figures 2a–2c). There were no significant differences in phototroph community structure between the soil surface and bulk soil incubated under light. NMDS analysis of TRFLP data showed two distinct clusters of samples: Grp I and Grp II (Figure 2a). Dark incubated samples were present in both Grp I and Grp II (Figures 2a–2b), however, all light incubated samples clustered within Grp II (Figure 2c), which suggests that phototroph community structure was more variable under dark compared to light conditions (Figures 2a–2c).

The soil surface incubated under light conditions had significantly different heterotrophic bacterial and fungal communities compared to bulk soil incubated under light and dark incubated samples (p≤0.01) (Figures 2d & 2e). There was no significant difference in heterotrophic bacterial and fungal community structure between bulk soil incubated under light and dark conditions (Figures 2d & 2e). At day 80, the soil surface harboured distinct bacterial communities under light conditions (Figure 2d).

### Microbial Community Structure and Taxonomic Diversity at the Soil Surface and in Bulk Soil after 80 days of Incubation

#### Phototroph community structure

**Figure 3:**
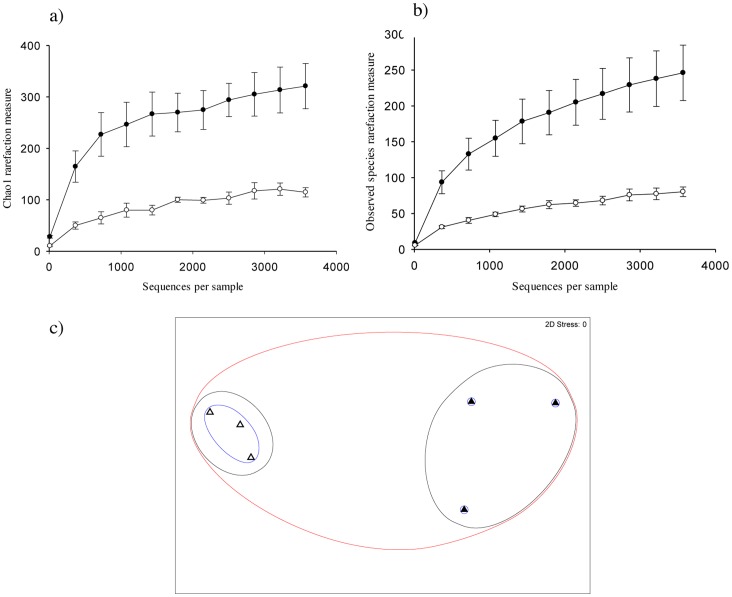
Phototroph diversity at the soil surface under light and dark conditions. α diversity estimates Chao1 (a) and Observed Species (b) and non-metric multidimensional scaling of community structure similarity (c) for phototrophs (23S rRNA genes of plastids) at the soil surface of a pasture soil after 80 days incubation under light (open symbols) or dark (closed symbols) conditions. OTU clustering was performed at the 97% similarity threshold using UCLUST. Error bars are ±1 S.E. Non-metric multidimensional scaling shows clustering based on the similarity of microbial community structure between treatments: 20% (red cluster), 25% (black cluster) and 80% (blue cluster).

Pyrosequencing revealed a total of 533 phototrophic OTUs across all samples with an average length of 351 bp, and an average of 71.7 reads assigned to each OTU, out of a total of 38203 processed reads. Chao1 index and Observed Species were both significantly higher at the soil surface incubated in the dark compared to light conditions (p≤0.001) (Figures 3a & 3b). Moreover, there were an estimated 246 unique phototroph OTUs under dark conditions compared to only 80 under light conditions (Figure 3b). Figures 3a and 3b both show that diversity plateaus under light as sampling depth increased, however, under dark conditions a plateau was not observed. NMDS analysis of phototroph community structure showed a closer clustering of samples under light compared to dark conditions, which suggests that phototroph community structure was less variable under light conditions (Figure 3c).

**Figure 4:**
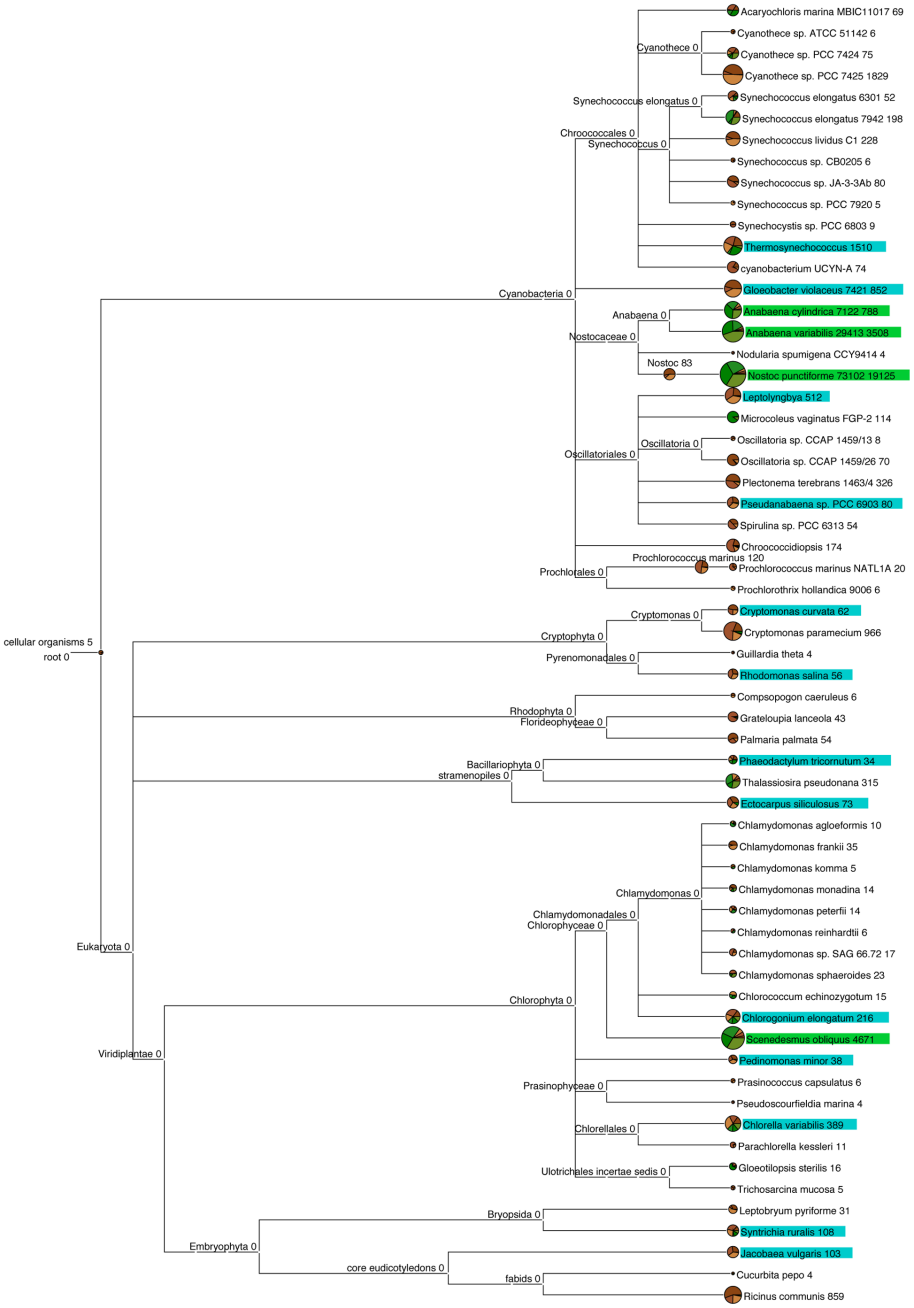
Phototroph community structure at the soil surface under light and dark conditions. The diversity and abundance of phototrophs (23S rRNA genes of plastids) at the soil surface of a pasture soil after 80 days incubation under light or dark conditions. Data is presented in MEGAN as an OTU table created in QIIME at a 97% similarity threshold (uclust). The number of reads that can be assigned to each taxon are shown at the end of each node. Pie charts show the proportion of reads assigned to each sample incubated under light (green) and dark (brown) conditions with replicates displayed as shades of these colours. Taxonomic assignments with only one read were removed. Significant differences in the read abundance of sequences between light and dark samples are highlighted in green when abundance is significantly higher under light conditions and in blue when abundance is significantly higher under dark conditions (p<0.05).

A wide range of cyanobacteria and eukaryotic phototrophs were detected, including green, red and brown algae, cryptomonads, diatoms, mosses, and angiosperms (Figure 4). Relative composition analysis showed that cyanobacteria, rather than eukaryotic phototrophs, dominated under both treatments, with a relatively greater number of reads assigned to cyanobacteria under light compared to dark conditions (p<0.01) (Table 2). Further, the relative composition of cyanobacteria differed between light treatments e.g. 65.1%±SE 0.96% and 12.6%±SE 2.17% of reads had close homology to *N. punctiforme* PCC 73102 under light and dark conditions, respectively (p≤0.001), 11.6%±SE 2.02% and 2.4%±SE 0.11% of reads had close homology to *Anabaena variabilis* ATCC 29413 under light and dark, respectively (p≤0.01), and 2.5%±SE 0.26% and 1.0%±SE 0.29% of reads had close homology to *A. cylindrica* PCC 7122 under light and dark, respectively (p≤0.05) (Figure 4). There were no clearly dominant taxa under dark conditions, rather, seven taxa had a relative read abundance between 6% and 15%, which ranked as follows: *Cyanothece* sp.>*N. punctiforme*>*Thermosynechococcus elongatus*>*Cryptomonas paramecium*>*Ricinus communis*>*Gloeobacter violaceus*>*Scenedesmus obliquus* (Figure 4).

**Table 2:** Relative read abundance of sequences with close homology to cyanobacteria and eukaryotic phototrophs from the soil surface of a pasture soil after incubation under light or dark conditions for 80 days (±1 standard error).

Taxonomy	Light (%)	Dark (%)
Cyanobacteria	63.8±3.36	82.7±2.03**
Eukaryotes	36.2±3.36	17.3±2.03**
- Green algae	12.8±1.76	15.8±1.89
- Red algae	1.1±0.036	0.01±0.00*
- Brown algae	0.67±0.08	0.03±0.02**
- Diatoms	1.1±0.21	0.93±0.24
- Cryptomonads	10.6±3.91	0.24±0.21*
- Mosses	1.1±0.18	0.10±0.03**
- Angiosperms	9.5±3.15	0.11±0.07*

Significant differences between light and dark treatments is indicated by a *(p≤0.05) or **(p≤0.01).

Relative composition analysis showed that a greater proportion of reads were assigned to eukaryotic phototrophs under dark compared to light conditions (p<0.001), in particular cryptomonads, red algae, brown algae, mosses and angiosperms (p<0.05) (Table 2). In contrast, relative composition analysis showed 6.2%±SE 1.25% and 14.8%±SE 1.88% of reads were assigned to *Scenedesmus obliquus* under dark and light conditions, respectively (p≤0.05). Relative composition analysis also showed a greater number of reads assigned to the green algae *Chlorella variabilis* (p≤0.05) and *Chlorogonium elongatum* (p≤0.05), brown alga *Ectocarpus siliculosus* (p≤0.001), moss *Syntrichia ruralis* (p≤0.05), angiosperm *Jacobaea vulgaris* (p≤0.001), diatom *Phaeodactylum tricornutum* (p≤0.05), and cryptomonads *Rhodomonas salina* (p≤0.001) and *Cryptomonas curvata* (p≤0.01) under dark compared to light conditions.

#### Bacterial community structure

**Figure 5:**
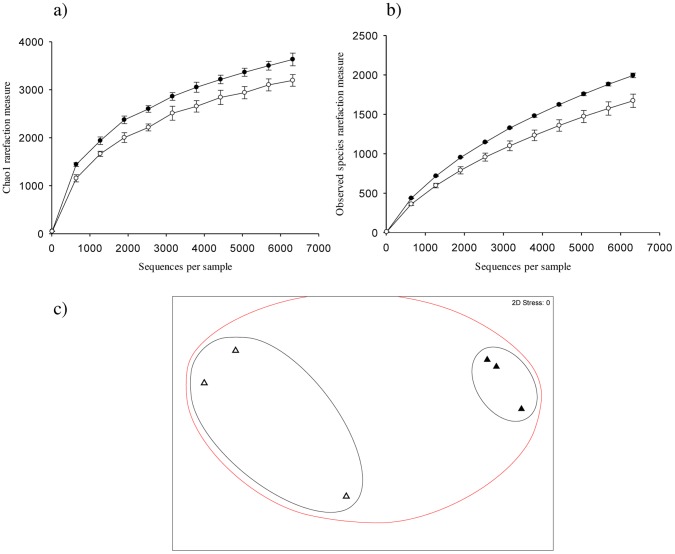
Bacterial diversity at the soil surface under light and dark conditions. α diversity estimates Chao1 (a) and Observed Species (b) and non-metric multidimensional scaling of community structure similarity (c) for bacteria (16S rRNA) at the soil surface of a pasture soil after 80 days incubation under light (open symbols) or dark (closed symbols) conditions. OTU clustering was performed at the 97% similarity threshold using uclust. Error bars are ±1 S.E. Non-metric multidimensional scaling shows clustering based on the similarity of microbial community structure between treatments: 45% (red cluster) and 55% (black cluster).

Analysis of pyrosequencing data for bacteria (49766 reads) clustered read data into 6517 bacterial OTUs with an average read length of 340 bp and an average of 7.6 reads assigned to each OTU. Chao1 index and Observed Species were significantly higher at the soil surface under dark compared to light conditions (p≤0.001) (Figures 5a & 5b). In contrast to phototrophs, NMDS analysis of bacterial community structure showed a closer clustering of dark compared to light incubated samples, which suggests that bacterial community structure was more variable at the soil surface under light conditions (Figure 5c).

**Figure 6:**
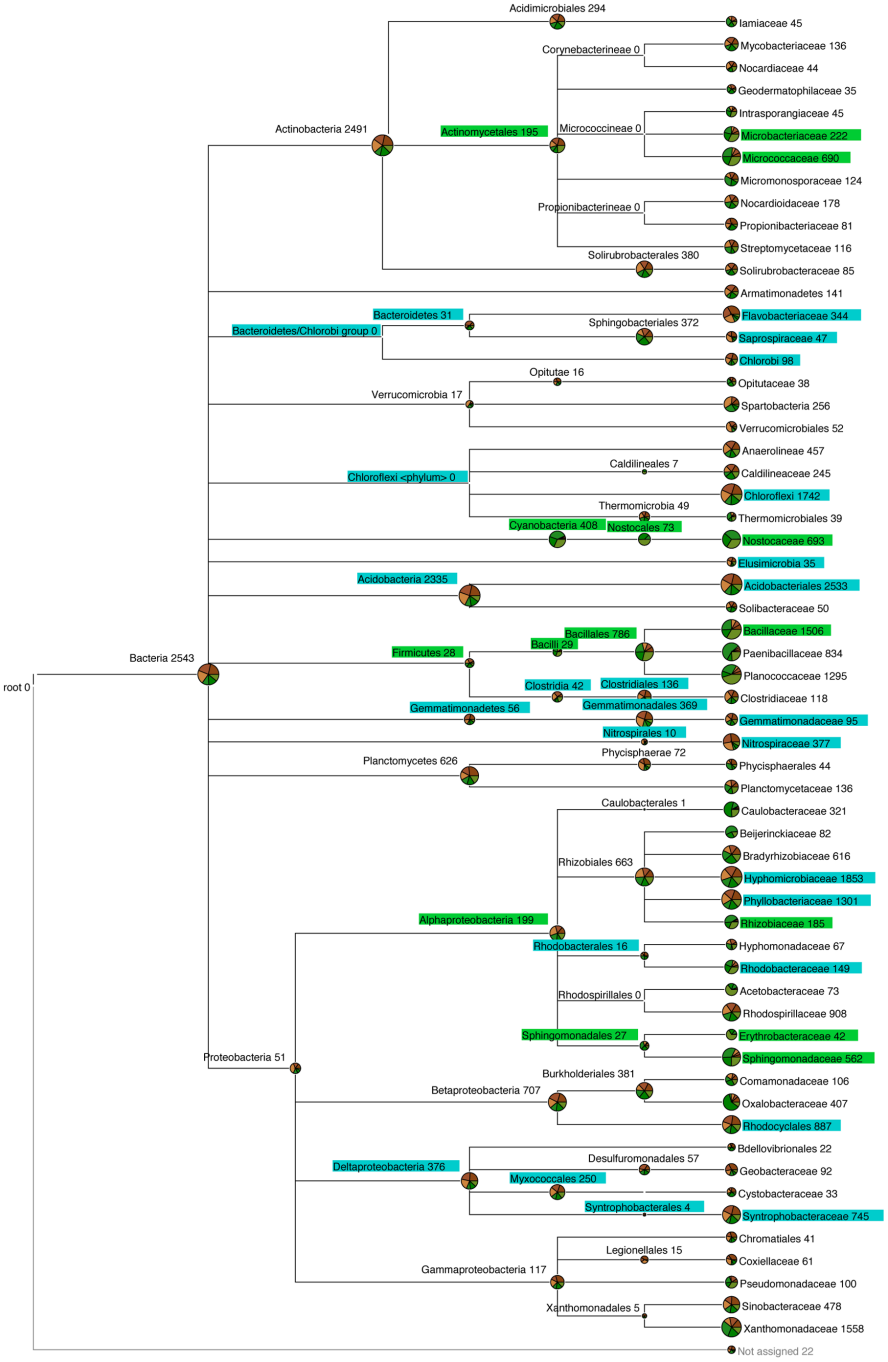
Bacterial community structure at the soil surface under light and dark conditions. The diversity and abundance of bacteria (16S rRNA gene) at the soil surface of a pasture soil after 80 days incubation under light or dark conditions. Data is presented in MEGAN as an OTU table created in QIIME at a 97% similarity threshold (uclust). The OTU table is presented at the taxonomic level of family. The number of reads that can be assigned using the RDP classifier at a confidence level of 80% are shown at the end of each node. Pie charts show the proportion of reads assigned to each sample incubated under light (green) and dark (brown) conditions with replicates shown as shades of these colours. Taxonomic assignments accounting for <0.5% total sequence abundance were removed. Significant differences in the read abundance of sequences between light and dark samples are highlighted in green when abundance is significantly higher under light conditions and in blue when abundance is significantly higher under dark conditions (p<0.05).

At the phylum level, relative composition analysis showed that Proteobacteria dominated the soil surface with 35.1%±SE 0.21% and 36.4%±SE 2.66% of reads assigned under dark and light conditions, respectively (Figure 6). The relative composition of samples showed that 19.3%±SE 4.39% and 5.9%±SE 0.18% of reads had close homology to the phylum Firmicutes under light and dark conditions, respectively (p≤0.05), and 5.9%±SE 1.21% and 2.0%±SE 0.03% of reads were assigned to the family Bacillaceae under light and dark conditions, respectively (p≤0.05) (Figure 6). Moreover, relative composition analysis showed that more reads were assigned to the class α-Proteobacteria (p≤0.05), the order Sphingomonadales (p≤0.001) and the families Sphingomonadaceae (p≤0.01) and Rhizobiaceae (p≤0.05) under light compared to dark conditions (Figure 6).

Relative composition analysis also showed that 5.4%±SE 0.14% and 3.0%±SE 0.04% of reads had close homology to δ-Proteobacteria under dark and light conditions, respectively (p≤0.01), and 2.5%±SE 0.02% and 1.4%±SE 0.3% of reads had close homology to Syntrophobacteraceae under dark and light conditions, respectively (p≤0.05) (Figure 6).

#### Fungal community structure

**Figure 7:**
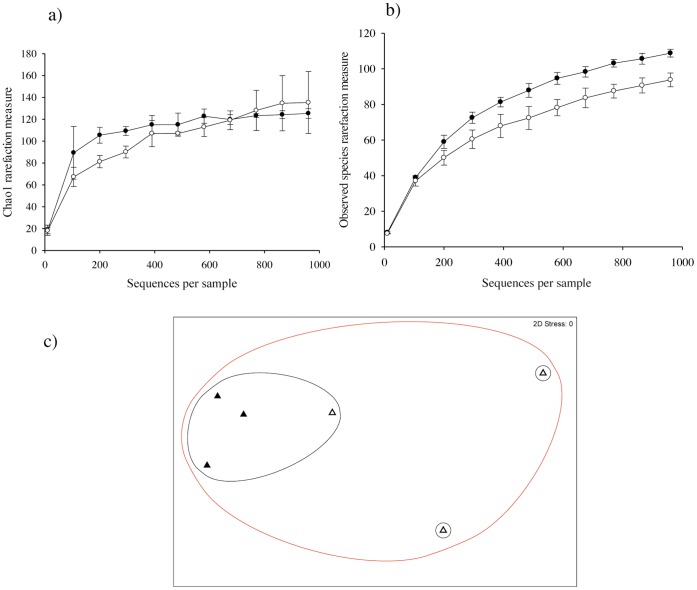
Fungal diversity at the soil surface under light and dark conditions. α diversity estimates Chao1 (a) and Observed Species (b) and non-metric multidimensional scaling of community structure similarity (c) for fungi (ITS region) at the soil surface of a pasture soil after 80 incubation under light (open symbols) or dark (closed symbols) conditions. OTU clustering was performed at the 97% similarity threshold using UCLUST. Error bars are ±1 S.E. Non-metric multidimensional scaling shows clustering based on the similarity of microbial community structure between treatments: 55% (red cluster) and 70% (black cluster).

Pyrosequencing (14577 reads) revealed 472 fungal OTUs with an average length of 316 bp and an average of 30.9 reads assigned to each OTU. However, Observed Species showed a significantly higher number of unique OTUs under dark compared to light conditions (p≤0.001) (Figures 7a & 7b). NMDS analysis of fungal community structure showed a poor clustering of light incubated samples under light conditions; one sample shared a greater similarity to dark incubated rather than light incubated samples, which suggests that fungal community structure was more variable under light compared to dark conditions (Figure 7c).

**Figure 8:**
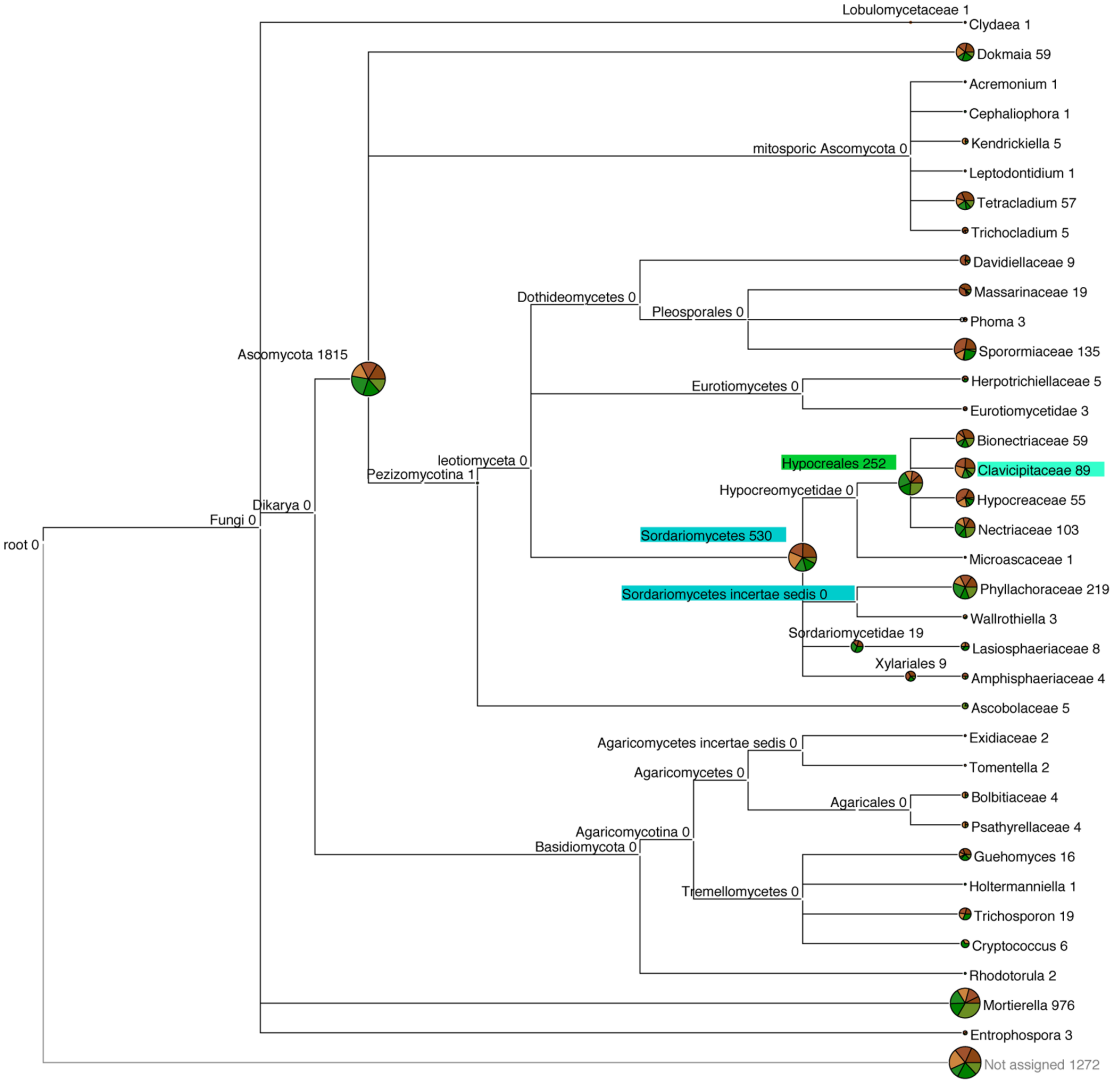
Fungal community structure at the soil surface under light and dark conditions. The diversity and abundance of fungi (ITS region) at the soil surface of a pasture soil after 80 days incubation under light or dark conditions. Data is presented in MEGAN as an OTU table created in QIIME at a 97% similarity threshold (uclust). The number of reads that can be assigned using the RDP classifier at a confidence level of 80% to each taxon are shown at the end of each node. Pie charts show the proportion of reads assigned to each sample incubated under light (green) and dark (brown) conditions with replicates shown as shades of these colours. Significant differences in the read abundance of sequences between light and dark samples are highlighted in green when abundance is significantly higher under light conditions and in blue when abundance is significantly higher under dark conditions (p<0.05).

Relative composition analysis showed Ascomycota to be the dominant division of fungi at the soil surface with 57.9%±SE 5.96% and 62.4%±SE 2.79% of reads showing close homology under light and dark conditions, respectively (Figure 8). The presence of light produced few shifts in fungal community structure, however, relative composition analysis showed that 2.3%±SE 0.09% and 4.3%±SE 0.53% of reads were assigned to Hypocreales under dark and light conditions, respectively (p≤0.05) (Figure 8). Relative composition analysis also showed a relatively greater number of reads assigned to both Sordariomycetes incertae sedis and Clavicipitaceae under dark compared to light conditions (p≤0.05) (Figure 8).

## Discussion

Light had a significant effect on phototroph community structure, soil nutrients and pH, and this effect extended to the underlying bulk soil. Light also had a significant, time-dependent impact on heterotrophic bacterial and fungal community structure which was restricted to the soil surface. Soil surface communities are typically defined by the presence of photosynthetic communities in the top 1–3 mm of soil [1], however, we show changes in phototroph community structure at a depth greater than 3 mm, and the presence of distinct heterotrophic microbial communities at the soil surface in the presence of light.

Chlorophyll *a* analysis was used as a broad-scale assessment of phototroph biomass development, and it indicated both the development of phototrophs at the soil surface after 20 days and the restriction of phototrophs to the soil surface under light conditions (Figure 1). The presence of light also significantly increased soil pH and reduced extractable NO_3_ and extractable Mg at both the soil surface and underlying bulk soil under light compared to dark conditions (Table 1). Therefore, although phototrophs appeared to be restricted to the soil surface, the influence of light extended to bulk soil (Figure 1; Table 1).

TRFLP analysis of phototrophs was used as a fine-scale assessment of community structure, and it showed development of distinct communities at the soil surface and bulk soil under light compared to dark incubated soil (Figures 2a–2c). In contrast to chlorophyll *a* data, TRFLP analysis showed no difference in phototroph community structure between the soil surface and underlying bulk soil under light conditions (Figures 2a–2c). Therefore, fine-scale molecular analysis has shown a new depth of influence of light on phototroph community structure that previous broad-scale assessments have missed [1]. It has previously been shown that approximately 0.3% of light is transmitted beyond the top 2 mm of soils with the highest transmittance of light [62]. Therefore, these shifts in phototroph community structure in bulk soil may be driven by attenuated light penetrating small cracks present at the soil surface. Alternatively, penetration of filamentous cyanobacteria into underlying soil may be a consequence of primary production at the soil surface under light conditions. These hypotheses require further testing, particularly in cracking clay soils where light penetration through soil cracks could result in significant shifts in phototroph community structure at even greater depths.

Distinct bacterial and fungal communities developed at the soil surface under light conditions compared to bulk soil, and dark incubated soil (Figures 2d & 2e). Although chlorophyll *a* data showed the development of phototrophs after only 20 days (Figure 1), shifts in bacterial and fungal communities were only evident after 40 days (Figures 2d & 2e). This time lag may be controlled by the time taken for light to indirectly affect soil pH and/or nutrient availability. The influence of light on bacterial and fungal communities was restricted to the soil surface which suggests they are either directly responding to light which is attenuated at lower depths, and/or indirectly responding to nutrients that are only altered at the soil surface, presumably as a result of the growth of phototrophs, such as extractable P or extractable K. Alternatively, heterotrophic bacterial and fungal communities may have an indirect response to availability of C fixed by phototrophs at the soil surface.

Light may also exert an additional indirect effect on community structure by elevating temperature and therefore accelerating the frequency of drying-rewetting cycles at the soil surface. It has previously been shown that drying-rewetting regimes can influence bacterial composition [63], [64] and fungal PLFA [65]. Placella *et al*. (2012) showed significant declines in the relative abundances of Actinobacteria and Acidobacteria, significant increases in the relative abundances of β- and γ- proteobacteria, and specific α-proteobacteria such as Sphingomonadales, and a bell-shaped response for Bacilli after soil re-wetting [64]. Relative composition analysis showed a similar effect of light on Bacilli and Sphingomonadales in the current study, which could be a consequence of more pronounced wet-dry cycles under light compared to dark conditions (Figure 6). However, it is important to note that Placella *et al*. (2012) investigated shifts in active communities over a short time-period (72-hour) after total soil water content was increased by ∼30% [64]. In contrast, weekly monitoring of soil moisture content in the current study showed water content did not differ by >1% between light and dark incubated samples.

Studies of the soil surface have typically focused on how bacterial and fungal communities differ based on geographical location, desert type, or aridity level; a direct impact of light on heterotrophic communities, however, has not been reported previously [6], [11], [36]. Moreover, we show community shifts between 40 and 80 days following a simulated tillage event, which adds to studies conducted under agricultural cropping systems, which have shown phototroph development between 50 and 80 days after tillage [15].

Phototroph diversity has been investigated using cultivation-dependent techniques [7], [9], [34], [32], [33] or molecular analysis targeting bacterial diversity in arid lands [2], [3], [6], [10], [11], [34], however, we reveal the diversity of both cyanobacteria and eukaryotic phototrophs at the soil surface of a temperate soil using 454 pyrosequencing. Using relative composition analysis, we show specific cyanobacterial taxa being selected for by light, namely *N. punctiforme*, *A. cylindrica* and *A. variabilis* (Figures 3–4; Table 2). The fact that relative composition analysis showed that significantly more reads were assigned to cryptomonads, red algae, brown algae, mosses, and angiosperms in the dark reflects that these proliferated less than cyanobacteria in the light but are nonetheless present in the seed bank of phototrophs (Figure 4; Table 2). The dominant cyanobacteria of BSCs has been shown to be influenced by several factors, including the type of BSC [3], successional stage [4], underlying soil substrata [2], and the level of aridity [11]. We show a selection for the diazotrophic cyanobacterium *N. punctiforme* at the surface of temperate soil, consistent with results documented in mature, or late-successional BSCs from arid lands [2], [4], [6], [7], [10], [34] (Figure 4). This suggests that diazotrophic cyanobacteria may also be important ecosystem engineers in temperate environments, in addition to arid zones [4], [20]–[24]. However, the contribution of surface communities to N_2_ fixation in temperate soils or agricultural systems remains to be elucidated. Such data could be beneficial for informing agricultural management decisions, for example, the realization that diazotrophs were able to fix an agriculturally significant proportion of N_2_ could influence decisions relating to soil tillage and the amount, frequency and timing of N fertiliser application under cropping systems.

454 pyrosequencing revealed that light also selected for heterotrophic bacteria at the soil surface (Figure 6). We found that in contrast to the desert soils studied to date [2], [3], [6], [11], few bacterial sequences (<4%) had close homology to cyanobacteria, allowing shifts in heterotrophic bacteria to be assessed (Figure 5–6). The comparative reduction in bacterial diversity under light conditions was not due to a selection for cyanobacteria (Figure 6; Table 2) as α diversity was still significantly lower under light conditions (p≤0.01) after the removal of photosynthetic bacterial OTUs from analysis (results not shown). The differences in diversity may be due to an input of C through photosynthesis and or N by N_2_ fixation, which could indirectly select for specific heterotrophic bacteria. This is analogous to the ‘rhizosphere effect.’ The rhizosphere is the area of soil under the influence of roots. Studies have shown that the rhizosphere can select for particular microbial communities and that this selection is plant-specific [66]. A similar effect may be occurring at the soil surface under light conditions. Moreover, taken with TRFLP results which show that the impact of light on bacterial community structure is restricted to the upper 3 mm of the soil surface (Figure 2c), a new research area of microbial influence may be emerging, which we term the ‘crustosphere.’

TRFLP and 454 pyrosequencing revealed that light also significantly impacted fungal community structure at the soil surface (Figure 2e & 8). The relatively few shifts in fungal communities could be due to the development stage of phototroph communities. BSCs typically undergo a succession from cyanobacteria- to lichen- to moss- dominated crusts in arid zones [32]. In the present study, the soil surface was dominated by cyanobacteria (Table 2). However, if the surface was left to develop to a lichen dominated community, more significant shifts in fungal community structure may be evident as lichen symbioses develop. However, parallels can still be drawn between soil surface fungal communities of temperate and arid lands, for example, relative composition analysis showed that Ascomycota were the dominant fungi in the present study in addition to surveys in the Colorado plateau, Chihuahuan desert and Sonoran deserts, USA [35]–[36].

In conclusion, the application of fine-scale molecular analysis gave new insights into soil surface community structure. We show differences in phototroph community structure in bulk soil in the presence of light, which have not previously been detected. We also show that the soil surface harbours distinct heterotrophic bacterial and fungal communities. Future work should focus on the ecological significance of both phototrophic and heterotrophic communities, particularly in temperate zones, including their functional importance in agro-ecosystems.

## Supporting Information

Figure S1 **Phototroph development at the soil surface.** Development of phototrophs at the surface of a pasture soil; (a) 9 days incubation under light conditions; (b) 14 days incubation under light conditions, and; (c) Comparison of dark and light incubated soil after 40 days incubation.
(TIF)

Figure S2 **Soil pH.** pH at the surface (▴) and bulk (▾) of pasture soil after incubation under light (open symbols) or dark (closed symbols) conditions. Errors bars are ±1 standard error.
(TIF)

Figure S3 **Soil nitrate.** Nitrate at the surface (▴) and bulk (▾) of pasture soil after incubation under light (open symbols) or dark (closed symbols) conditions. Errors bars are ±1 standard error.
(TIF)

Figure S4 **Soil potassium.** Potassium at the surface (▴) and bulk (▾) of pasture soil after incubation under light (open symbols) or dark (closed symbols) conditions. Errors bars are ±1 standard error.
(TIF)

Figure S5 **Soil magnesium.** Magnesium at the surface (▴) and bulk (▾) of pasture soil after incubation under light (open symbols) or dark (closed symbols) conditions. Errors bars are ±1 standard error.
(TIF)

Figure S6 **Soil phosphorous.** Phosphorous at the surface (▴) and bulk (▾) of pasture soil after incubation under light (open symbols) or dark (closed symbols) conditions. Errors bars are ±1 standard error.
(TIF)

Table S1 Soil properties of Gartenacker topsoil (10–20 cm) taken from Switzerland.
(DOCX)

Table S2 Primer pairs used to investigate bacterial, fungal and phototroph community structure in Gartenacker soil incubated under light and dark conditions.
(DOCX)

Table S3 Number of phototroph sequences removed at each processing step.
(DOCX)

Table S4 Number of bacterial sequences removed at each processing step.
(DOCX)

Table S5 Number of fungal sequences removed at each processing step.
(DOCX)

Supporting Information S1 (DOCX)
