# Supplementary figures and images for: Light Structures Phototroph, Bacterial and Fungal Communities at the Soil Surface
Source: PLoS One. 2013 Jul 19;8(7):e69048. doi: 10.1371/journal.pone.0069048 (PMC3716809; doi:10.1371/journal.pone.0069048)

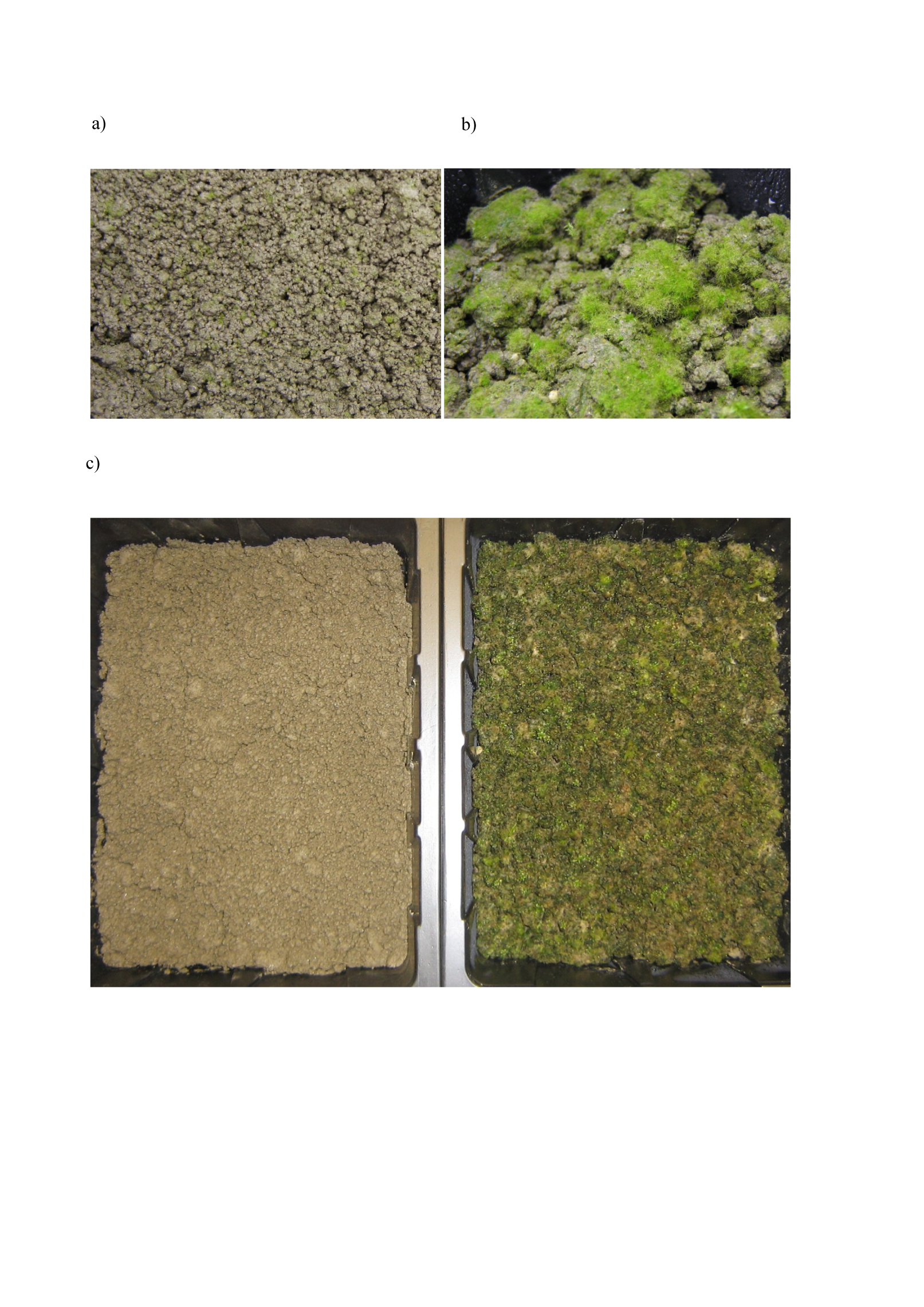

Supplement: Figure S1 — Phototroph development at the soil surface. Development of phototrophs at the surface of a pasture soil; (a) 9 days incubation under light conditions; (b) 14 days incubation under light conditions, and; (c) Comparison of dark and light incubated soil after 40 days incubation. (TIF) [file pone.0069048.s001.tif]

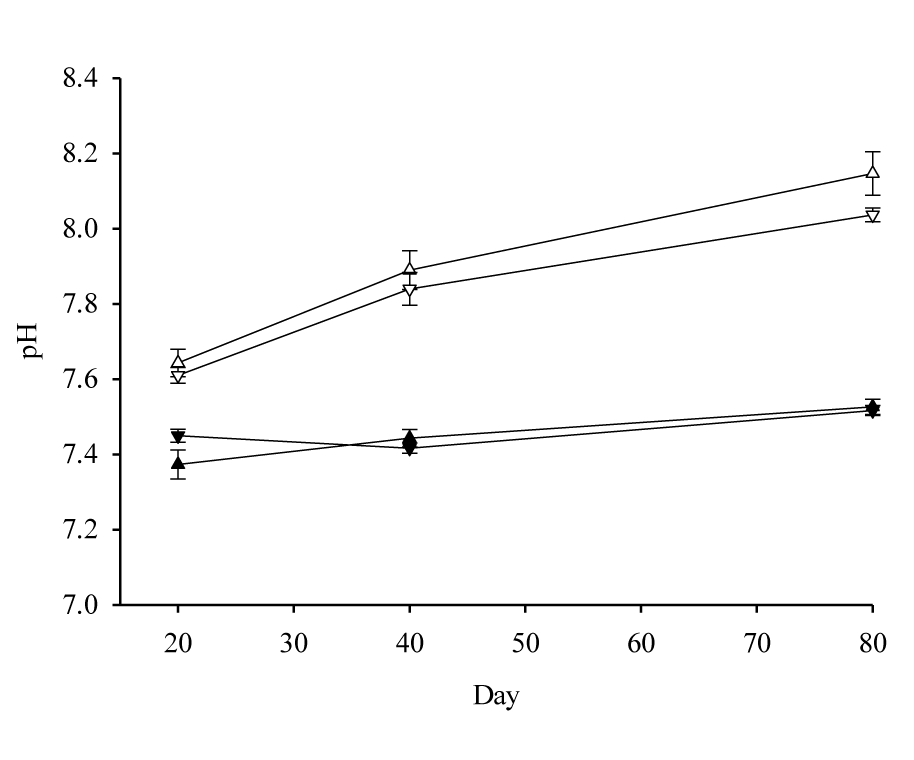

Supplement: Figure S2 — Soil pH. pH at the surface (▴) and bulk (▾) of pasture soil after incubation under light (open symbols) or dark (closed symbols) conditions. Errors bars are ±1 standard error. (TIF) [file pone.0069048.s002.tif]

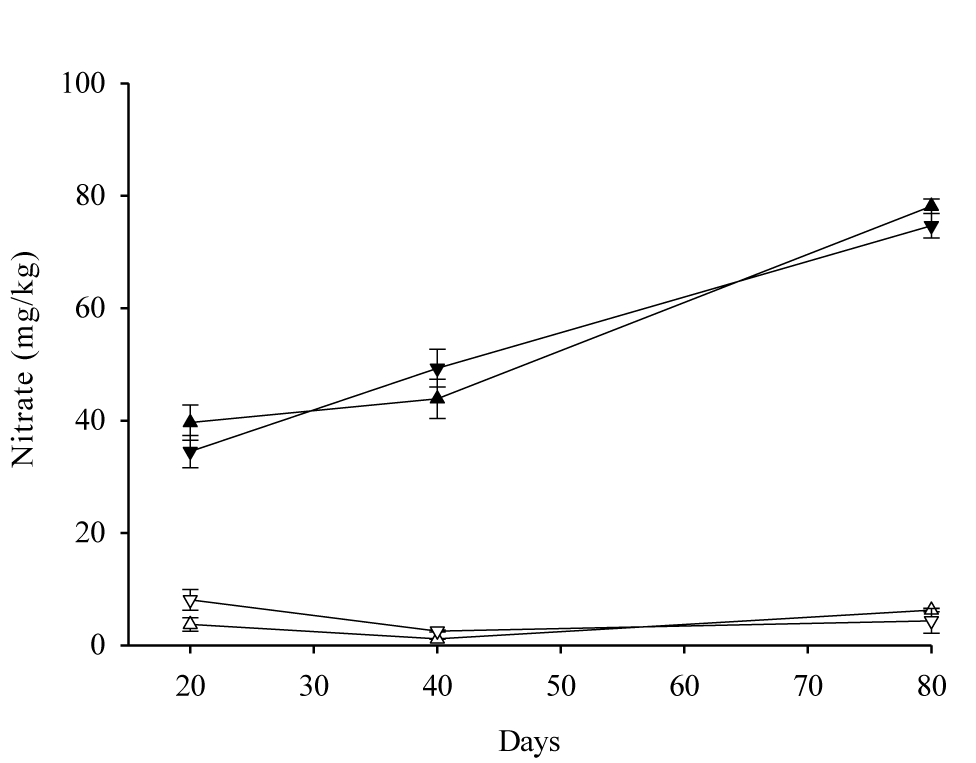

Supplement: Figure S3 — Soil nitrate. Nitrate at the surface (▴) and bulk (▾) of pasture soil after incubation under light (open symbols) or dark (closed symbols) conditions. Errors bars are ±1 standard error. (TIF) [file pone.0069048.s003.tif]

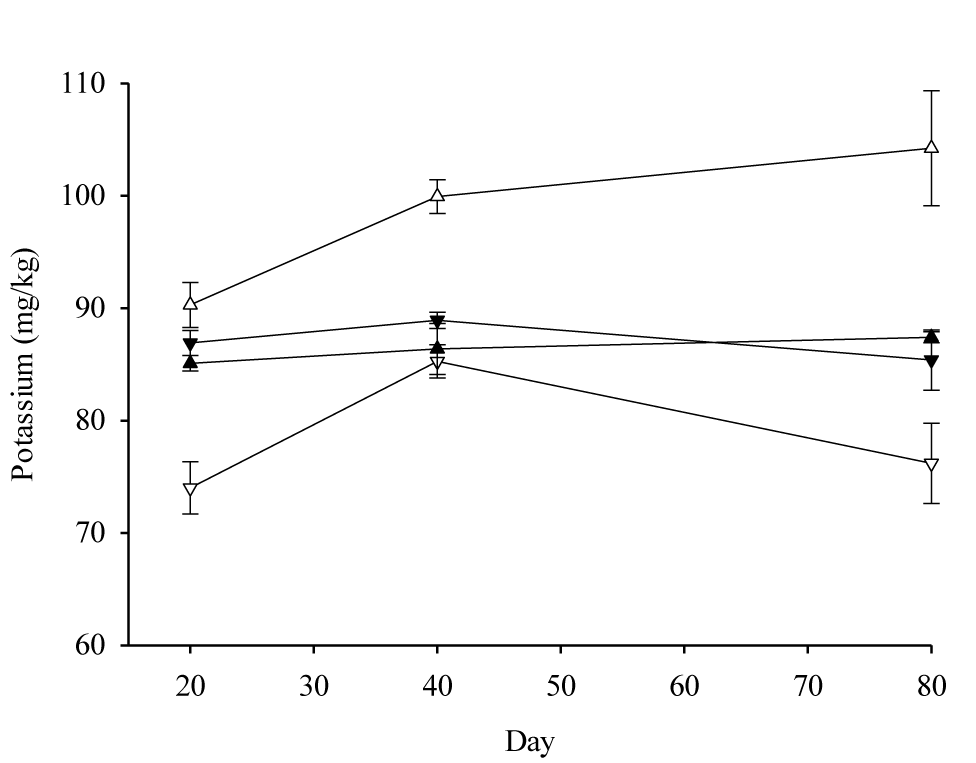

Supplement: Figure S4 — Soil potassium. Potassium at the surface (▴) and bulk (▾) of pasture soil after incubation under light (open symbols) or dark (closed symbols) conditions. Errors bars are ±1 standard error. (TIF) [file pone.0069048.s004.tif]

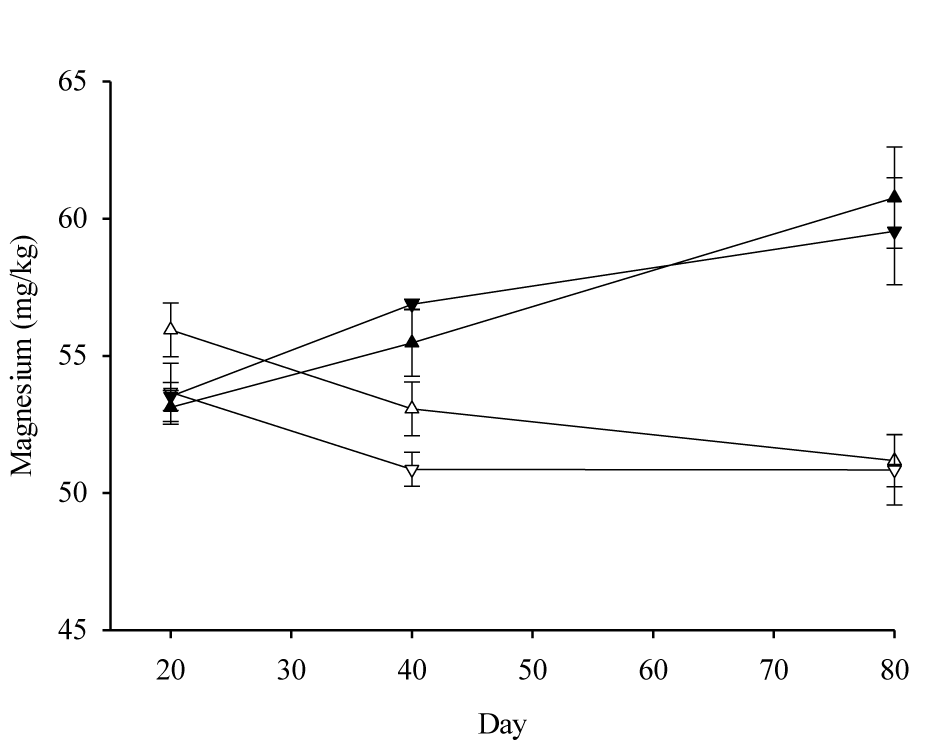

Supplement: Figure S5 — Soil magnesium. Magnesium at the surface (▴) and bulk (▾) of pasture soil after incubation under light (open symbols) or dark (closed symbols) conditions. Errors bars are ±1 standard error. (TIF) [file pone.0069048.s005.tif]

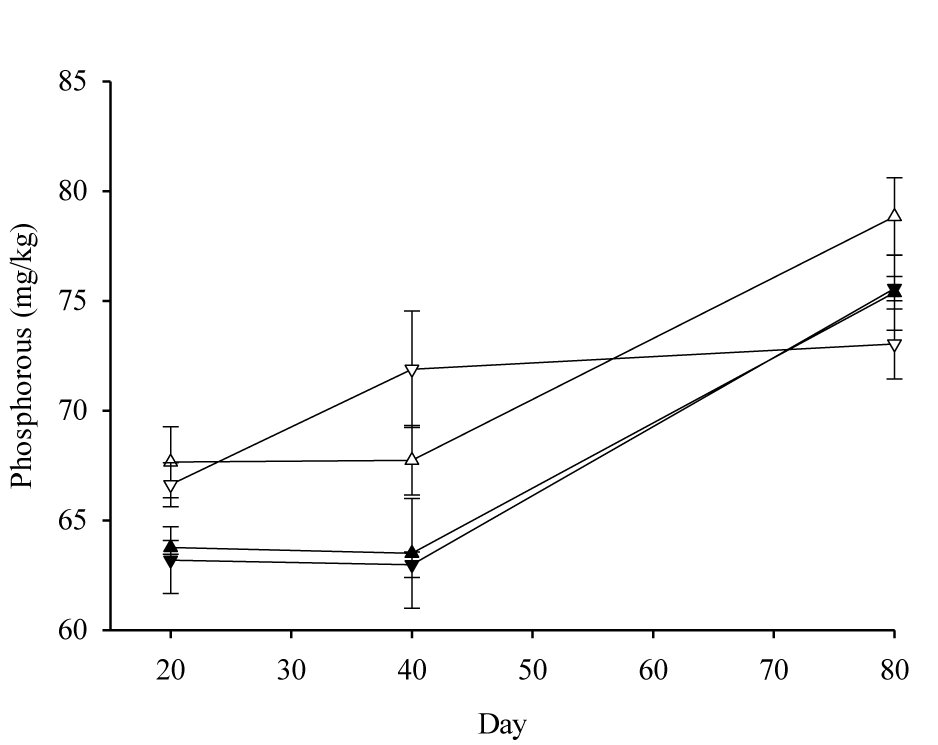

Supplement: Figure S6 — Soil phosphorous. Phosphorous at the surface (▴) and bulk (▾) of pasture soil after incubation under light (open symbols) or dark (closed symbols) conditions. Errors bars are ±1 standard error. (TIF) [file pone.0069048.s006.tif]
