# Supplementary material for: Light Structures Phototroph, Bacterial and Fungal Communities at the Soil Surface
Source: PLoS One. 2013 Jul 19;8(7):e69048. doi: 10.1371/journal.pone.0069048 (PMC3716809; doi:10.1371/journal.pone.0069048)
Supplement: Table S1 — Soil properties of Gartenacker topsoil (10–20 cm) taken from Switzerland. (DOCX) [file pone.0069048.s007.docx]

**Table S1: Soil properties of Gartenacker topsoil (10-20 cm) taken from Switzerland**

| Soil | Classification | pH | | % OM | CEC | Particle size analysis (%) | | | Moisture holding capacity (%) | |
| --- | --- | --- | --- | --- | --- | --- | --- | --- | --- | --- |
|  |  |  | |  |  |  | | |  |  |
|  |  | H_2_O | 0.01M CaCl_2_ |  | meq/100g | Sand | Silt | clay | 1/3 bar | 15 bar |
| Gartenacker | Silt loam | 6.9 | 6.6 | 4.1 | 10.1 | 34 | 52 | 14 | 29.6 | 16.0 |
