# Supplementary material for: Light Structures Phototroph, Bacterial and Fungal Communities at the Soil Surface
Source: PLoS One. 2013 Jul 19;8(7):e69048. doi: 10.1371/journal.pone.0069048 (PMC3716809; doi:10.1371/journal.pone.0069048)
Supplement: Table S2 — Primer pairs used to investigate bacterial, fungal and phototroph community structure in Gartenacker soil incubated under light and dark conditions. (DOCX) [file pone.0069048.s008.docx]

**Table S2: Primer pairs used to investigate bacterial, fungal and phototroph community structure in Gartenacker soil incubated under light and dark conditions**

| Organism | Region | Primer sequence | Fragment size (bp) | Restriction enzyme |
| --- | --- | --- | --- | --- |
| Algae | 23S | **p23SrV_R1-HEX** (5′TCAGCCTGTTATCCC TAGAG 3′)  **p23SrV_f1** (5′GGACAGAAAGACCCT ATGAA 3’) | 410 | *Dde*I |
| Bacteria | 16S | **63f** (5′AGGCCTAACACATGCAA GTC3’)  **1087r-VIC** (5′CTCGTTGCGGGACTTACC CC3′) | 1000 | *Hha*I, *Msp*I |
| Fungi | ITS | **ITS1F-PET** (5′CTTGGTCATTTAGAGGAAGTAA3’)  **ITS4r** (5′TCCTCCGCTTATTGATAT GC3’) | 750 | *Hha*I, *Msp*I |
