# Supplementary material for: Light Structures Phototroph, Bacterial and Fungal Communities at the Soil Surface
Source: PLoS One. 2013 Jul 19;8(7):e69048. doi: 10.1371/journal.pone.0069048 (PMC3716809; doi:10.1371/journal.pone.0069048)
Supplement: Table S3 — Number of phototroph sequences removed at each processing step. (DOCX) [file pone.0069048.s009.docx]

**Table S3: Number of phototroph sequences removed at each processing step**

|  | Raw data | Split libraries | Chimera removal | Non-phototrophs^1^ |
| --- | --- | --- | --- | --- |
| No. seqs | 77 470 | 50 962 | 46 281 | 38 203 |
| Minimum No. seqs | 8 558 | 5 292 | 4 814 | 3 317 |
| Maximum No. seqs | 16 223 | 11 365 | 10 256 | 10 188 |
| Mean No seqs | 12 912 | 8 494 | 7 714 | 6 367 |

^1^ The non-phototroph processing step refers to the removal of all representative sequences that shared close homology with heterotrophic bacteria
