# Supplementary material for: Light Structures Phototroph, Bacterial and Fungal Communities at the Soil Surface
Source: PLoS One. 2013 Jul 19;8(7):e69048. doi: 10.1371/journal.pone.0069048 (PMC3716809; doi:10.1371/journal.pone.0069048)
Supplement: Table S4 — Number of bacterial sequences removed at each processing step. (DOCX) [file pone.0069048.s010.docx]

**Table S4: Number of bacterial sequences removed at each processing step**

|  | Raw data | Split libraries | Chimera removal |
| --- | --- | --- | --- |
| No. seqs | 67 658 | 54 767 | 49 766 |
| Minimum No. seqs | 8 536 | 6 853 | 6 322 |
| Maximum No. seqs | 13 552 | 11 059 | 9 834 |
| Mean No seqs | 11 276 | 9 127 | 8 294 |
