# Supplementary material for: Light Structures Phototroph, Bacterial and Fungal Communities at the Soil Surface
Source: PLoS One. 2013 Jul 19;8(7):e69048. doi: 10.1371/journal.pone.0069048 (PMC3716809; doi:10.1371/journal.pone.0069048)
Supplement: Table S5 — Number of fungal sequences removed at each processing step. (DOCX) [file pone.0069048.s011.docx]

**Table S5: Number of fungal sequences removed at each processing step**

|  | Raw data | Split libraries | Chimera removal |
| --- | --- | --- | --- |
| No. seqs | 22 672 | 15 141 | 14 577 |
| Minimum No. seqs | 1 574 | 1 012 | 964 |
| Maximum No. seqs | 5 469 | 3761 | 3 647 |
| Mean No seqs | 3 779 | 2 523.5 | 2430 |
