# Supplementary material for: Light Structures Phototroph, Bacterial and Fungal Communities at the Soil Surface
Source: PLoS One. 2013 Jul 19;8(7):e69048. doi: 10.1371/journal.pone.0069048 (PMC3716809; doi:10.1371/journal.pone.0069048)
Supplement: Supporting Information S1 — (DOCX) [file pone.0069048.s012.docx]

**Supporting Information**

*Bold’s basal media*

Bold’s Basal Media was produced by adding the following stock solutions to 940ml d. H_2_O: 10 ml NaNO_3_ (25 g l^-1^), 10 ml CaCl_2_.2H_2_O (2.5 g l^-1^), 10 ml MgSO_4_.7H_2_O (7.5 g l^-1^), 10 ml K_2_HPO_4_ (7.5 g l^-1^), 10 ml KH_2_PO_4_ (17.5 g l^-1^), 10 ml NaCl (2.5 g l^-1^), 1 ml EDTA (50 g l^-1^) and KOH (31 g l^-1^), 1 ml FeSO_4_.7H_2_O (4.98 g l^-1^) with 1 ml H_2_SO_4_, 1 ml H_3_BO_3_ (11.42 g l^-1^), and 1 ml micronutrients solution (ZnSO_4_.7H_2_O (8.82 g l^-1^) MnCl_2_.4H_2_O (1.44 g l^-1^), MoO_3_ (0.71 g l^-1^), CuSO_4_.5H_2_O (1.57 g l^-1^), and Co(NO_3_)_2_.6H_2_O (0.49 g l^-1^)).

*PCR reactions*

The PCR reactions used to assess phototroph, bacterial and fungal community structure are shown below:

Phototroph samples were run on a GeneAmp 9700 thermocycler under the following conditions: initial denaturation at 94^o^C for 2 mins, followed by 35 cycles of 94^o^C for 20 secs, an annealing step at 55^o^C for 30 secs and extension at 72^o^C for 30 secs, before a final extension period at 72^o^C for 10 mins.

Fungal and bacterial samples were run under the following conditions: initial denaturation at 95^o^C for 3 mins, followed by 30 cycles of 95^o^C for 30 secs, 55^o^C for 1 min and 72^o^C for 1 min, before a final extension period at 72^o^C for 10 mins.
